# The thrombophilic network of autoantibodies in celiac disease

**DOI:** 10.1186/1741-7015-11-89

**Published:** 2013-04-04

**Authors:** Aaron Lerner, Nancy Agmon-Levin, Yinon Shapira, Boris Gilburd, Sandra Reuter, Idit Lavi, Yehuda Shoenfeld

**Affiliations:** 1 Pediatric Gastroenterology and Nutrition Unit, Carmel Medical Center, B. Rappaport School of Medicine, Technion-Israel institute of Technology, Michal St. No 7, Haifa, 34362, Israel; 2 The Zabludowicz Center for Autoimmune Diseases, Sheba Medical Center, Tel-Ashomer, 52621, Israel; 3 Aira e.v./Aesku.Kipp Institute, Mikroforum Ring 3, Wendelsheim, 55234, Germany; 4 Carmel Medical Center, Epidemiology and Community Medicine, Michal St. No 7, Haifa, 34362, Israel

**Keywords:** Antiphosphatidylserine/prothrombin, Autoantibodies, Celiac disease, Hypercoagulability, Phospholipid, Prothrombin

## Abstract

**Background:**

Celiac disease is a life-long autoimmune condition, affecting genetically susceptible individuals that may present with thromboembolic phenomena. This thrombophilia represents a puzzle with multiple constituents: hyperhomocysteinemia, B12 and\or folate deficiency, methylenetetrahydrofolate reductase mutations, and protein C and S deficiency due to vitamin K deficiency. However, the well known thrombogenic factors, antiphosphatidylserine/prothrombin and antiprothrombin have never been explored in celiac disease.

**Methods:**

The serum autoantibody levels were determined in 248 individuals, classified into three groups. Group 1 comprised 70 children with definitive celiac disease (age: 7.04 ±4.3 years, male to female ratio 1.06) and group 2 comprised 88 normal children (age: 6.7 ±4.17 years, male to female ratio 0.87), representing controls. The pediatric populations were compared to group 3, which included 90 adults who were family members (parents) of group 1 (age: 34.6 ±11.35 years, male to female ratio 1.2). Antibodies were checked by enzyme-linked immunosorbent assay.

**Results:**

Mean optical density levels of serum antiphosphatidylserine/prothrombin immunoglobulin G antibodies were 32.4 ±19.4, 3.6 ±2.5 and 16.1 ±15.8 absorbance units in groups 1, 2 and 3 respectively (*P* <0.0001), with 45.7%, 0% and 7.8% of groups 1, 2 and 3 respectively positive for the antibody (*P* <0.01). Mean optical density levels of serum antiphosphatidylserine/prothrombin immunoglobulin M antibodies were 14.2 ±8.7, 6.7 ±6.4 and 12.4 ±15.5 absorbance units in groups 1, 2 and 3 respectively (*P* <0.0001), with 7.1%, 3.4% and 9.9% of groups 1, 2 and 3 positive for the antibody. Mean optical density levels of serum antiprothrombin and antiphospholipid immunoglobulin G antibodies were higher in groups 1 and 3 compared with 2 (*P* <0.005) and in groups 1 and 2 compared with 3 (*P* <0.01), respectively. Groups 1, 2 and 3 were positive for antiphospholipid immunoglobulin G antibodies (groups 1 and 2 compared with 3) . Celiac disease sera harbor a higher antiprothrombin immunoglobulin G level compared with controls.

**Conclusions:**

It is suggested that the intestinal injury, endothelial dysfunction, platelet abnormality and enhanced apoptosis recently described in celiac disease are at the origin of the increased exposure of phospholipids or new epitopes representing autoantigens. Those autoantibodies might play a pathogenic role in the thrombophilia associated with celiac disease and represent markers for potential anticoagulant preventive therapy.

## Background

Celiac disease (CD) is the most common autoimmune food intolerance in the world. It is a life-long autoimmune condition [1] mainly of the gastrointestinal tract, affecting the small intestine of genetically susceptible individuals. Environmental factors are crucial for disease induction. Gluten, which is the storage protein of wheat and its alcohol-soluble gliadins are the offending inducers of the disease together with structurally related molecules found in barley, rye and oat. Tissue transglutaminase (tTG) is the autoantigen against which the abnormal immune response is directed [2] and two main autoantibodies, antiendomysium and anti-tTG, are currently the most useful serological markers to screen for the disease [3,4]. The sequential chain of events operating in the disease was recently unraveled, and gives hope for future therapeutic strategies [5]. Furthermore, the epidemiology, prevalence and clinical presentation of CD are changing constantly and, with time, new clinical presentations are depicted that increase the clinical variability of CD [6].

It has been shown that the classical intestinal clinical picture of malnutrition, chronic diarrhea and nutritional deficiencies are disappearing and extraintestinal presentations are emerging. Skin, endocrine, skeletal, hepatic, hematological, gynecological, fertility, dental and behavioral abnormalities are often described [7-9]. Nowadays, we are witnessing an epidemiological shift in the disease phenotype toward a more advanced age, and increased prevalence of latent, hyposymptomatic or asymptomatic presentations [6].

A newly explored area of CD is hypercoagulability and the resulting thromboembolic phenomena. There is an increase risk of stroke in adults and children with CD [10-15]. Thrombophilia, pregnancy loss, deep vein thrombosis, small bowel infarction, atrial fibrillation, Budd-Chiari syndrome, portal and splenic vein thrombosis, and cardiovascular disease have been described [16-21]. Even the onset of the disease may be due to a thrombotic event [11,17,21]. Hyperhomocysteinemia with related vitamin deficiency in untreated CD, the frequency of methylenetetrahydrofolate reductase variants and the high homology between factor XIII and tTG add to the hypercoagulable status in patients [21-26].

In fact, there is an increased incidence of autoimmune diseases in CD [1,7,27,28]. Two examples associated with thrombophilia are systemic lupus erythematosus (SLE) and antiphospholipid (aPL) syndrome [29,30]. Three autoantibodies associated with the two entities are antiphosphatidylserine/prothrombin (aPS/PT), aPL and antiprothrombin (aPT). aPS/PT and aPL autoantibodies confer increased risk for thromboembolic events and poor outcome in those diseases [31-39]. The correlation between aPS/PT antibodies and clinical manifestations of aPL syndrome and the importance of aPS/PT as a marker for this syndrome are well established. The relationship between aPS/PT antibodies and hypercoagulability state is further strengthened by their increased incidence in cerebral infarction [40]. aPT autoantibodies are prevalent in SLE and aPL syndrome and are associated with thrombosis and pregnancy morbidity [41-44].

Despite the coexistence of CD and thromboembolic events, the aPS/PT and aPT status has never been investigated and aPL activity has scarcely been investigated in CD. On the above backgrounds of aPS/PT, aPT and aPL antibodies and thrombophilia, hypercoagulability in CD, and increased incidence of SLE and aPL in CD, the presence of aPS/PT, compared with aPL, aPT and anticardiolipin antibodies, were explored in children with CD and their parents, compared with pediatric controls. Increased incidence of aPS/PT IgG in the celiac group and intermediate incidence in their parents, compared with none in the control group, was detected. Additionally, higher rates of activities of aPS/PT IgM and prothrombin IgG autoantibodies in the celiac patients compared with the other two groups were detected. It seems that the presently studied thrombophilic autoantibodies are operative in CD, extending the hypercoagulability network in this disease.

## Methods

### Study populations

Serum aPS/PT, aPT and aPL autoantibodies levels were determined in 248 individuals, divided into three groups. Group 1 comprised 70 Israeli children with definitive CD (age 7.04 ±4.3 years, male to female ratio 1.06). Group 2 was represented by 88 normal children (age 6.7 ±4.17 years, male to female ratio 0.87) as controls. The pediatric populations were compared with group 3, which included 90 family members (parents) of group 1 (age 34.6 ±11.35 years, male to female ratio 1.2).

The following information was collected on the three groups: diet - gluten-containing or gluten-free; symptoms - abdominal pain, short stature, vomiting, diarrhea, anemia, failure to thrive and IgA deficiency; familial diseases - CD, diabetes mellitus type 1 or 2, familial Mediterranean fever, inflammatory bowel disease, thyroid disease; laboratory parameters - complete blood count, biochemical profile, IgA levels, CD serology (see ELISA assays below).

CD was diagnosed according to the revised criteria of the European Society for Pediatric Gastroenterology and Nutrition, based on specific serology and duodenal biopsies [45]. All the participants were on a gluten-containing diet and were checked for celiac serology.

### ELISA assays

#### Celiac serology

Three ELISA assays are included in our celiac screening algorithm, as recently described [4,46]. Briefly, The AESKU celiCheck (Aesku.Kipp Institute, Wendelsheim, Germany) determines IgA and IgG neo-tTG antibodies, those antibodies against the new epitopes created in the transformed complex of gliadin-tTG. In this study, we evaluated The AESKU CeliCheck Neo-epitope assay on the TRITURUS analyzer (GRIFOLS SA, Barcelona Spain). All the participants were additionally screened for tTG IgA assay on the Liaison (DiaSorin, Saluggia, Italy) and the ORGENTEC tTG IgA plus IgG assay on the ETI-MAX 3000 analyzer (DiaSorin). National external quality assessment site is routinely used as the external quality control program.

#### Anticardiolipin, phospholipid, prothrombin and aPS/PT essays

Sera were tested for anticardiolipin, phospholipid, prothrombin and aPS/PT antibodies using solid phase enzyme immunoassay (AESKULISA, AESKU diagnostics ( Aesku.Kipp Institute, Wendelsheim, Germany) , according to the manufacturer’s protocol.

Briefly, serum samples were diluted 1:100 and incubated in microplates coated with the specific antigen. Binding was detected by antihuman immunoglobulins peroxidase (conjugate) and 3,3',5,5'-Tetramethylbenzidine-substrate. The sera was identified as positive for the antibodies according to the manufacture's equations for cut-off value determination or using other cut-offs as specified below:

**Cardiolipin check -** The immunoassay employed highly purified cardiolipin plus native human beta2-cardiolipin 1 for the combined quantitative and qualitative detection of IgA, IgM and IgG antibodies against cardiolipin in the sera. Positive cut-off was >24 U/ml.

**Phospholipid IgG and IgM -** As for the cardiolipin check except for the use of antihuman IgG and IgM peroxidise. Positive cut-off for both antibodies was >18 U/ml.

**Prothrombin IgG -** The immunoassay employed highly purified prothrombin (factor ІІ) for the combined quantitative and qualitative detection of IgG antibodies against prothrombin in the sera. Antihuman IgG peroxidase conjugate was employed. Positive cut-off was >18 U/ml.

**Phosphatidylserine/prothrombin IgA, IgG and IgM -** The immunoassay employed highly purified phosphatidylserine plus native human prothrombin for the combined quantitative and qualitative detection of IgA, IgM and IgG antibodies against PS/PT in the sera. Antihuman IgA, IgG and IgM peroxidase conjugates were used. Positive cut-off for PS/PT -IgA was >28 U/ml. The manufacturers cut-off is 18 U/ml. Based on multiple determinations on 92 healthy Israeli participants, a higher cut-off of the mean plus two SD was used. The receiver operating characteristic curve data were: area under the curve 0.855; standard error 0.0315; 95% confidence interval 0.791, 0.905; Z statistic 11.258; and *P* <0.0001.

### Endoscopy and intestinal histology

All patients in group 1 underwent esophagogastro-duodenoscopy using a GIF-xp 20 endoscope (Pentax, Tokyo, Japan). At least five biopsies were obtained: four from the second part of the duodenum for the diagnosis or exclusion of CD and one from the antrum.

The biopsies were immediately fixed in buffered formalin and embedded on edge in paraffin. Sections were stained with hematoxylin-eosin and Giemsa, analyzed by the pathologist and graded according to Marsh criteria, as previously described [3]. On the day of endoscopy, 5 ml of peripheral blood was withdrawn, centrifuged at 5000 c/s for 10 minutes, and the serum frozen in −80° Celsius until assayed for serology.

The ethical committee of Carmel Medical Center approved the study and written informed consent was obtained from the parents or guardians of the children.

### Statistical analysis

Data analysis was performed using the PASW 18 statistical package (PASW, Chicago, IL, USA). A comparison of the levels of anticardiolipin, phospholipid, prothrombin and aPS/PT autoantibodies between the three study groups was performed by a Kruskal-Wallis test. For multiple comparisons between any two study groups, a Mann Whitney test was used. For examining the association between the positive cut-offs for all antibodies with the study groups, a Chi square test or exact test for small sample was used. All *P* values were two-sided, and statistical significance was defined as *P* <0.05.

## Results

No epidemiological statistical difference between the pediatric groups (groups 1 and 2) was detected. None of the participants were IgA deficient and all were screened also by IgG-tTG antibodies. None of the parents (group 3) at the time of the study had positive serology for CD, despite consuming gluten. No correlation was found between parents and children concerning the results.

Table 1 shows the mean ±SD and median of the different autoantibodies in group 1 (pediatric CD), group 2 (pediatric control) and the parents of group 1. Table 2 shows the mean of percentage positivity of the optical density of the autoantibodies in group 1 (pediatric CD) and group 3 (parents) compared with the healthy controls, group 2.

**Table 1:** Mean and median of autoantibodies' activity in celiac children, their parents compared to pediatric controls

**Mean ± SD (median)**						**Autoantibodies/groups**
**Parents versus pediatric control**	**Pediatric celiac disease versus pediatric control**	**Pediatric celiac disease versus parents**	**Pediatric control (N = 88)**	**Parents (N = 90)**	**Pediatric celiac disease (N = 70)**	
ND	ND	**	ND	10.7 ±13.6 (8.2)	7.9 ±9.5 (5.6)	aPS/PT IgA
**	**	**	3.6 ±2.5 (3.3)	16.1 ±15.9 (12.3)	32.4 ±19.5 (27.7)	aPS/PT IgG
**	**	**	6.7 ±6.4 (4.9)	12.4 ±15.5 (8.6)	14.2 ±8.7 (12.9)	aPS/PT IgM
*	**	*	11.9 ±15.8 (6.0)	12.4 ±18.2 (7.9)	15.2 ±19.5 (10.6)	Prothrombin IgG
**	**	**	9.4 ±7.0 (8.6)	8.0 ±10.4 (5.8)	10.4 ±6.0 (9.1)	Phospholipid IgG
ND	ND	*	ND	3.9 ±6.9 (1.7)	3.3 ±2.8 (2.5)	Phospholipid IgM
NS	NS	**	5.5 ±3.4 (4.9)	2.9 ±2.1 (2.2)	4.5 ±2.6 (4.4)	Cardiolipin check

*P <0.001; ** *P* <0.0001; aPL: antiphospholipid; aPS/PT: antiphosphatidylserine/prothrombin; aPT: antiprothrombin; Ig: immunoglobulin; ND: not done NS: non-significant; SD: standard deviation.

**Table 2:** Percentage positivity of autoantibodies in celiac children and their parents compared with pediatric controls

***P***	**Pediatric controls (N = 88)**	**Celiac disease parents (N = 90)**	**Pediatric celiac disease (N = 70)**	**Optical density cut-off levels**	**Autoantibodies positivity/groups**
<0.9	ND.	3.3	4.3	>28	aPS/PT IgA
<0.0001	0***,**	***7.8, **	45.7***	>28	aPS/PT IgG
0.23>	3.4	9.9	7.1	>28	aPS/PT IgM
0.5>	14.9	11	17.1	>18	Prothrombin IgG
0.07>	*12.5	*3.3	11.4	>18	Phospholipid IgG
1>	ND.	2.2	1.4	>18	Phospholipid IgM
1>	0	1.1	0	>24	Cardiolipin check

*P <0.05,**P <0.01,***P <0.0001; aPS/PT: antiphosphatidylserine/prothrombin; aPT: antiprothrombin; Ig: immunoglobulin.

## Discussion

The main result of the present study is the increased incidence of aPS/PT IgG in the celiac group and intermediate incidence in their parents, compared with none in the control group. Secondary results are the increased rates of aPS/PT IgM and prothrombin IgG autoantibodies in the celiac patients compared with the other two groups. Of note is the constant, parallel, gradual decrease of the levels of aPS/PT IgG and IgM across the continuum of the three groups, from celiac children, to their parents, to pediatric controls. The fact that none of the parents had positive celiac serology points to a potential genetic influence on the presence of aPS/PT autoantibodies. In fact, being an autoimmune disease with a well-established genetic susceptibility and increased familial predisposition, the increased presence of autoantibodies and autoimmune diseases in first-degree relatives of CD patients is well known [47-49] and aPS/PT should be added to the list. Additionally, aPS/PT should be added to the increasing list of autoantibodies associated with CD-affected patients [27,28].

Despite the fact that many of the autoantibodies might present an epiphenomenon, it is suggested that mainly aPS/PT, but also antithrombin and aPL autoantibodies, are pathogenic and play an active role in CD pathogenesis and complications. The presence of aPS/PT is directly related to thromboembolic events in aPL syndrome, SLE and cerebral infarction [31-40]. The thrombogenic properties of aPS/PT correlate with increased thrombin generation in aPL syndrome, contributing to the understanding of the pathophysiology of thrombophilia in these patients [36]. Those autoantibodies are strong risk factors for venous thromboembolism in patients with SLE because they induce activated protein C resistance [37]. The other two IgG autoantibodies, namely antithrombin and aPL, are also associated with thrombotic events in aPL syndrome and SLE [32,33,50,51], and are risk factors for myocardial infarction in middle-aged men [52,53].

The pathophysiology of the thromboembolic phenomena associated with CD [10-26] represents a puzzle with multiple constituents: hyperhomocysteinemia; B12 and\or folate deficiency; methylenetetrahydrofolate reductase mutations; the high homology between blood coagulation factor XIII and tTG; and protein C and S deficiency due to vitamin K deficiency [21-26,54]. The present study unravels a series of autoantibodies, aPL, aPT and mainly aPS/PT, that form part of this puzzle and are suggested to play a pathogenic role in the thrombogenicity of CD.

Phosphatidylserine is a regular constituent of the inner leaflet of the cell membrane, which is only exposed on the outside of the cell membrane during apoptosis or because of damaged endothelial cells [55]. It is known that prothrombin and aPL antibodies bind specifically to the surface of apoptotic cells [56,57]. Recently, Ieko *et al.*[58] reported that aPS/PT IgG recognizes prothrombin bound to phosphatidylserine on platelets and endothelial cells and, directly or via Fc-gamma receptors, activates a variety of procoagulant agents. However, the complementary aspects of CD are endothelial dysfunction [59], platelet abnormalities [60,61] and increased apoptosis [62]. Thus, it is suggested that intestinal injury, endothelial dysfunction, platelet abnormalities and enhanced apoptosis cause increased exposure of phospholipids or new epitopes, which are the origin of aPT, aPL and aPS/PT autoantibodies. Those antibodies might play a pathogenic role in the thrombophilia associated with CD.

New light was recently shed on the ‘inflammation coagulation crosstalks’ [63]. Recent studies have unveiled molecular underpinnings of the intimate interconnection between both systems. Being a classical inflammatory state, CD can present such crosstalks, resulting in enhanced coagulability in the intestinal arena and on the systemic level. Because there are several pathways of mucosal injury, autoantigens like phospholipids, phosphatidylserine and prothrombin are exposed, inducing the production of aPS/PT, aPL and aPT antibodies. With their thrombogenic capacities, those autoantibodies can present the first or an additional hit in the thrombogenic background operating in CD. Because of the increased coagulability in CD and the harmful potential consequences, patients positive for those antibodies should be considered to receive preventive anticoagulant therapy.

## Conclusions

We detected increased incidence of aPS/PT IgG in the pediatric celiac group and intermediate incidence in their parents, compared with no incidence in the control group. Additionally, higher rates of activity for aPS/PT IgM and prothrombin IgG autoantibodies were observed in the patients with CD compared with the other two groups. Based on the extensive literature of thromboembolic phenomenon described in CD, it seems that the thrombophilic autoantibodies studied here are operative in CD, extending the hypercoagulability network of the disease. The use of the autoantibodies described in this study as potential markers for thromboembolic manifestations in CD is a subject for future exploration.

## Abbreviations

aPL: Antiphospholipid; aPS/PT: Antiphosphatidylserine/prothrombin; aPT: Antiprothrombin; CD: Celiac disease; ELISA: Enzyme-linked immunosorbent assay; Ig: Immunoglobulin; SD: Standard deviation; SLE: Systemic lupus erythematosus; tTG: Tissue transglutaminase

## Competing interests

The authors declare that they have no competing interests.

## Authors’ contributions

AL, NA-L and YSho conceived of the study, and participated in its design and coordination and drafted the manuscript. YSha and BG carried out all the immunoassays. SR supervised the laboratory analysis and directed the information processing. IL analyzed the data and performed the statistics. All authors read and approved the final manuscript.

## Pre-publication history

The pre-publication history for this paper can be accessed here:

http://www.biomedcentral.com/1741-7015/11/89/prepub
